# Pre-pregnancy obesity is not associated with poor outcomes in fresh transfer in vitro fertilization cycles: a retrospective study

**DOI:** 10.1186/s12884-023-05917-7

**Published:** 2023-09-02

**Authors:** Ping Tao, Xiaohong Yan, Yan Yao, Zhanxiang Wang, Youzhu Li

**Affiliations:** 1 Reproductive Medicine Centre, First Affiliated Hospital of Xiamen University, 55# Zhenhai Road, Xiamen, Fujian 361000 P.R. China; 2 Department of Epidemiology and Health Statistics, School of Public Health, Jilin University, 1163# Xinmin Street, Changchun, Jilin 130021 P.R. China; 3 Department of Neurosurgery, First Affiliated Hospital of Xiamen University, 55# Zhenhai Road, Xiamen, Fujian 361000 P.R. China

**Keywords:** Body mass index, In vitro fertilization, Embryo quality, Clinical outcomes, Obesity

## Abstract

**Purpose:**

The impact of body mass index (BMI) on in vitro fertilization (IVF) has been well acknowledged; however, the reported conclusions are still incongruent. This study aimed to investigate the effect of BMI on IVF embryos and fresh transfer clinical outcomes.

**Methods:**

This retrospective cohort analysis included patients who underwent IVF/ICSI treatment and fresh embryo transfer from 2014 to March 2022. Patients were divided into the underweight group: BMI < 18.5 kg/m^2^; normal group: 18.5 ≤ BMI < 24 kg/m^2^; overweight group: 24 ≤ BMI < 28 kg/m^2^; and obesity group: BMI ≥ 28 kg/m^2^. A generalized linear model was used to analyze the impact of BMI on each IVF outcome used as a continuous variable.

**Results:**

A total of 3465 IVF/ICSI cycles in the embryo part; and 1698 fresh embryo transplanted cycles from the clinical part were included. Available embryos rate (61.59% vs. 57.32%, *p* = 0.007) and blastocyst development rates (77.98% vs. 66.27%, *p* < 0.001) were higher in the obesity group compared to the normal BMI group. Also, the fertilization rate of IVF cycles in the obesity group was significantly decreased vs. normal BMI group (normal: 62.95% vs. 66.63% *p* = 0.006; abnormal: 5.43% vs. 7.04%, *p* = 0.037), while there was no difference in ICSI cycles. The clinical outcomes of overweight and obesity groups were comparable to the normal group. The gestational age of the obesity group was lower compared to the normal group (38.08 ± 1.95 vs. 38.95 ± 1.55, *p* = 0.011). The adjusted OR (AOR) of BMI for the preterm birth rate of singletons was 1.134 [(95% CI 1.037–1.240), *p* = 0.006]. BMI was significantly associated with live birth rate after excluded the PCOS patients [AOR: 1.042 (95% CI 1.007–1.078), *p* = 0.018]. In young age (≤ 35 years), clinical pregnancy rate and live birth rate were positively correlated with BMI, AOR was 1.038 [95% CI (1.001–1.076), *p* = 0.045] and 1.037 [95% CI (1.002–1.074) *p* = 0.038] respectively.

**Conclusion:**

Being overweight and obese was not associated with poor IVF outcomes but could affect blastocyst formation. ICSI could help to avoid low fertilization in obese patients. Also, obesity was associated with increased rates of premature singleton births.

**Supplementary Information:**

The online version contains supplementary material available at 10.1186/s12884-023-05917-7.

## Introduction

Obesity is a severe global health issue associated with hypertension, diabetes, as well as cardiovascular and cerebrovascular diseases. With the development of the economy and the improvement in living conditions, the proportion of obese people has rapidly increased, currently, over 2 billion people worldwide are overweight or obese accounting for one-third of the total population [1]. This proportion would be higher in European and North American developed countries [2].

Body mass index (BMI) is commonly used to measure the degree of obesity. According to the China Obesity working group, BMI is divided into underweight (< 18.5 kg/m^2^), normal (18.5–23.9 kg/m^2^), overweight (24–27.9 kg/m^2^), and obese categories (≥ 28 kg/m^2^) [3]. For females, the rise in BMI is often accompanied by sex hormone disorders and dyslipidemia, resulting in polycystic ovary syndrome (PCOS), menstrual dysfunction, ovulation disorders, and insulin resistance, which may affect reproductive functions [4, 5]. This may also have a negative impact on in vitro fertilization (IVF) outcomes (e.g., lower implantation rate, pregnancy, live birth rate [6, 7], higher cycle cancellation, and abortion rate [8]). BMI also affects the quality of oocytes and embryos. Increased chromosome abnormalities in the oocyte have been found in obese mice [9]. Fawarseh et al*.* examined the effect of maternal BMI on embryo morphokinetics using a time-lapse incubator and the effect on outcomes of frozen embryo transfer cycles, finding significantly faster oocyte cleavage at t3 and t5-t8 in underweight and overweight patients compared to those with normal weight [10]. Contrary, some other studies reported different results on the effect of BMI on IVF results [11, 12]. For example, Bellver et al*.* analyzed 3316 ICSI cycles and found that blastocyst formation and embryo morphokinetics were similar among different BMI groups [13]. Another study stated that clinical pregnancy and miscarriage rates were comparable between normal weight and overweight women [14]. Inconsistent data between studies may be due to the number of recruited cycles, the progress in ovulation induction technology, the different population, and statistical methods. In addition, some studies suggested an association between age and BMI, i.e., women < 35 years old were more likely to have higher BMI [15, 16]. Nevertheless, these studies mainly focused on the inter-group analysis of BMI rather than the analysis of BMI as a continuous variable.

In the current study, we conducted a group analysis based on BMI and an analysis with BMI as a continuous variable to investigate the effect of BMI on IVF embryo quality and fresh transfer clinical outcomes. In addition, we analyzed the effect of BMI in different age groups.

## Materials and methods

### Study design

This study included infertile patients who received IVF/ICSI treatment in the reproductive center of the First Affiliated Hospital of Xiamen University between October 2014 and March 2022. The First Affiliated Hospital Ethics Committee of Xiamen University (No.2022097) provided ethical approval for this retrospective study. A total of 4127 IVF/ICSI cycles were achieved during this period. The research of this study consisted of two parts, i.e., embryo outcomes and clinical outcomes. In embryo outcomes, the following cycles were excluded: canceled for early ovulation cycles (*n* = 18), ICSI cycles which did not inseminate for no mature oocytes (*n* = 19), no oocyte retrieved cycles (*n* = 76), ten frozen-oocyte cycles, incomplete embryo information cycles (*n* = 145), rescue-ICSI cycles (*n* = 158), complete fertilization failure cycles (*n* = 179) and cycles with missing BMI data (*n* = 57). The clinical outcomes analysis was based on 1753 fresh embryo transfer cycles from October 2014 to August 2021. Repeated failure cycles (*n* = 48) and 7 incomplete clinical outcome cycles were excluded in this part. Clinical outcomes were the primary outcomes in this study.

Finally, 3465 IVF/ICSI cycles in the embryo outcome part, 1698 fresh embryo transplanted cycles, and 813 delivery cycles involved in these cycles in the clinical outcome part were included in the analysis. The enrolled patients were divided into the underweight group: BMI < 18.5 kg/m^2^; normal group: 18.5 ≤ BMI < 24 kg/m^2^; overweight group: 24 ≤ BMI < 28 kg/m^2^; and obesity group: BMI ≥ 28 kg/m^2^. Embryo outcome indicators included: number of oocytes retrieved, mature oocytes rate, IVF/ICSI 2PN rate, IVF/ICSI (1 +  ≥ 3PN) rate, cleavage rate, available embryos rate, good quality embryos rate, blastocyst development rate and good quality blastocyst rate. Pregnancy outcome-related indicators included: ET embryo number, transferred embryo stage, at least one top-quality embryo transferred rate, endometrial thickness, clinical pregnancy rate, implantation rate, abortion rate, deliveries (singleton or twins), live birth rate. And perinatal outcome indicators included: gestational age, sex ratio, preterm birth rate, birthweight, Low birthweight rate (LBW), high birthweight rate (HBW), small for gestational age (SGA), very small for gestational age (VSGA), large for gestational age (LGA). These indicators were compared with normal groups by chi-square test. The above indicators were also included in the analysis of BMI as a continuous variable, this part of the comparison used regression analysis and adjusted confounding factors that may affect the outcomes. Pre-pregnancy and pregnancy information were collected before or during treatment, while abortion or perinatal-related information was collected by phone call follow-up.

### Ovarian stimulation for fresh IVF/ICSI cycles

Most of the stimulation cycles were treated by the gonadotropin-releasing hormone (GnRH) agonist protocol (60.20%, 2086 cycles) or antagonist protocol (22.66%, 785 cycles); the rest underwent progestin-primed ovarian stimulation (PPOS) protocol (12.47%, 432 cycles) and natural cycles (4.68%, 162 cycles).

GnRH agonist protocol was performed as previously described [17]. In the antagonist protocol, gonadotrophin (Gn) was given to start ovulation induction on the 2^nd^—4^th^ day of the menstrual cycle according to the patient's age, BMI, and ovarian reserve. Also, cetrorelix acetate (Merck Serono, Germany) 0.25 mg was added when the average follicle diameter was about 12 ∼ 14 mm. When the diameter of at least 2 dominant follicles was ≥ 18 mm, or the diameter of 4 follicles was ≥ 17 mm, human chorionic gonadotrophin (hCG, Aize, Germany Merck Seranol Co., Ltd.) 0.25 mg or 0.25 mg hCG + 0.2 mg of triptorelin acetate (Dabijia, Germany Ferring Pharmaceutical Co., Ltd.) were administered.

The PPOS protocol was performed as follows: from the 2^nd^ to 4^th^ day of the menstrual cycle, Gn was injected intramuscularly, and medroxyprogesterone acetate (MPA) (Zhejiang Xianju Pharmaceutical) was given orally for 10 mg / d, QD until the day of trigger.

### Laboratory procedure and embryo assessment

Oocyte retrieval was performed by follicular puncture under the guidance of vaginal B-ultrasound, and all follicles with a diameter ≥ 10 mm were extracted. Conventional IVF or ICSI was performed according to the patient's situation. Embryos were cultured in an incubator at 37 °C in an atmosphere of 6%CO2. Some 16–20 h after insemination, on day 1 (D1), fertilization was identified. Double pronuclei (2pn) was normal fertilization; single pronucleus (1PN) and ≥ 3 pronuclei fertilization were abnormal fertilization. On day 3 (D3), the embryo was scored according to the size, morphology, and fragment ratio of cleavage. The good quality embryo was defined when D1 fertilization was normal, with cleavages ≥ 6, and the proportion of fragments was < 20%; the available embryo was defined when D1 fertilization was normal, with cleavages ≥ 6 embryo cleavages, and the proportion of fragments was < 40%. According to Gardner's scale, Embryos that developed to the blastocyst stage were evaluated [18]. Good quality blastocysts included blastocysts (stage 3–6) with A or B scores for inner cell mass and trophectoderm.

### Embryo transfer and outcome measures

Embryo transfer was performed on day 3 or day 5 based on the specific situation of patients. The procedures were described in our previous research [17]. After oocyte retrieval, a progesterone injection of 60 mg was given once a day. Blood β-hCG was tested 12–14 days after embryo transfer; those with confirmed biochemical pregnancy continued receiving progesterone support until 26–28 days after embryo transfer. Those with one of the following conditions were considered clinically pregnant: (1) with gestational sac; (2) the pulsation of the primitive heart tube seen under ultrasound; (3) abortion, ectopic pregnancy history, and pregnancy confirmed by pathological examination. Abortion was defined as the termination of pregnancy with gestational age < 28 weeks, and premature birth was defined as live births with a gestational age ˃ 28 weeks and < 37 weeks.

### Statistical analysis

SPSS19.0 was used for statistical analysis. Continuous variables are expressed as mean ± standard deviation (‾x ± s). For continuous variables, the Shapiro–Wilk test was used to test for data normality and Kruskal–Wallis 1-way ANOVA (k samples) was used for analysis between different groups. Categorical variables are expressed as percentages (%), and a chi-square test was applied to compare the differences between groups. In the analysis of intra-group results, dichotomous variables such as pregnancy rate, abortion rate, and live birth rate were analyzed by binary logistic regression, expressed by relative risk (OR) and 95% CI. Continuous variables such as oocyte retrieval rate, fertilization rate, available embryo rate, good-quality embryo rate, and gestational age were analyzed by a generalized linear model expressed by beta (SE). Significant covariates and confounding factors were adjusted. In addition, factors that may affect embryo quality and clinical outcomes were screened. The variables finally included in the analysis of embryonic outcome were: age of men and women, infertility type (primary or secondary), infertility factors (fallopian tube factors, male factors, endometriosis, PCOS, unknown cause), infertility years, fertilization methods (IVF or ICSI), ovulation promotion program, basic endocrine (FSH, LH). Body mass index (BMI) was a continuous variable in all analyses. In order to visualize the relationship between BMI and clinical pregnancy rate and live birth rate, the (non-linear) logistic regression method was used to study confounders involved in these two dependent variables, which have been mentioned above. The OR value and its 95% confidence interval were calculated. Next, women were grouped according to their age, after which the same treatment was carried out. The curve was drawn using the "ggplot2" package. The whole process of drawing was implemented in the R language software (R.4.2.1). A *p*-value < 0.05 was considered statistically significant.

## Results

### Comparison of baseline characteristic data and embryo outcome of patients

A total of 3465 cycles were retrospectively analyzed in the embryo outcome part, including 385 cycles in the underweight group, 2194 cycles in the normal group, 708 cycles in the overweight group, and 178 cycles in the obesity group. The age of the total study cohort was (32.78 ± 5.55) years, and the BMI was (22.10 ± 3.25) kg/m2. The details of patient baseline characteristics according to maternal BMI are shown in Table 1. From the perspective of the whole patient group, with BMI increasing, the infertility year increased, whereas the basal FSH/LH/ E2 decreased (all *p* < 0.001). Compared with the normal group, patients in the underweight group were younger, unlike patients in the overweight group who were older (30.91 ± 5.47 *p* < 0.001 and 33.69 ± 5.96 *p* = 0.001, respectively, vs. 32.80 ± 5.38). Furthermore, the proportion of primary infertility was higher in the underweight group and lower in the overweight group (65.97% *p* < 0.001 and 42.37% *p* = 0.004, respectively, vs. 48.63% in the normal group). The rate of endometriosis decreased with increasing BMI, and the prevalence of PCOS significantly rose when BMI exceeded normal levels. In addition, the amount of AFC and total Gn dose among the four groups also increased with BMI, ranging from 12.52 ± 6.84 to 15.51 ± 9.84 and 1687.09 ± 638.87IU to 2630.26 ± 970.72IU, respectively (all *p* < 0.001). After excluding PCOS patients, only the AFC of the obesity group was higher than that of the normal group, but there was still no significant difference in the number of oocytes retrieved between groups (Table 2). Also, there was no difference in mature oocyte rate, ICSI fertility rate, total cleavage rate, and good quality embryo rate on D3 among the groups (all *p* > 0.05, Table 2).

**Table 1:** Baseline characteristics of the study cycles

Parameters	BMI < 18.5	18.5 ≤ BMI < 24	24 ≤ BMI < 28	BMI ≥ 28	*P*^*a*^	*P*^*b*^	*P*^*c*^
TOTAL: *n* = 3465	*n* = 385 (11.11%)	*n* = 2194 (63.32%)	*n* = 708 (20.42%)	*n* = 178 (5.14%)			
Maternal age (years)	30.91 ± 5.47	32.80 ± 5.38	33.69 ± 5.96	32.73 ± 5.26	< 0.001	0.001	0.266
BMI (kg/m2)	17.58 ± 0.72	21.14 ± 1.48	25.53 ± 1.11	30.15 ± 2.17	< 0.001	< 0.001	< 0.001
Infertility years	3.36 ± 2.45	3.65 ± 3.07	3.81 ± 2.77	3.90 ± 3.18	< 0.001	< 0.001	< 0.001
Total dose of GN (IU)	1687.09 ± 638.87	1947.50 ± 757.15	2211.21 ± 876.27	2630.26 ± 970.72	< 0.001	< 0.001	< 0.001
Basal FSH (mIU/ml))	7.37 ± 2.46	7.63 ± 3.03	7.27 ± 2.87	6.39 ± 2.34	< 0.001	< 0.001	< 0.001
Basal LH (mIU/ml)	5.30 ± 2.22	4.95 ± 2.21	4.82 ± 2.54	4.71 ± 2.67	< 0.001	0.001	< 0.001
Basal E2 (pg/ml)	53.91 ± 20.77	48.62 ± 19.63	46.14 ± 18.62	45.84 ± 18.93	< 0.001	< 0.001	< 0.001
AFC	12.52 ± 6.84	12.74 ± 7.84	13.67 ± 8.21	15.51 ± 9.84	< 0.001	< 0.001	< 0.001
AFC (excluded PCOS patients)	10.56 ± 6.75	10.76 ± 7.40	11.30 ± 7.69	12.55 ± 8.97	0.603	0.119	0.024
Type of infertility (%)							
primary infertility	65.97(254/385)	48.63(1067/2194)	42.37(300/708)	48.88(87/178)	< 0.001	0.004	0.938
secondary infertility	34.03(131/385)	51.37(1127/2194)	57.63(408/708)	50.56(90/178)			
Main infertility factor (%)							
Tubal factor	43.12(166/385)	42.39(930/2194)	42.09(298/708)	35.39(63/178)	0.823	0.896	0.07
Endometriosis	16.36(63/385)	10.26(225/2194)	8.19(58/708)	3.93(7/178)	< 0.001	0.095	0.054
PCOS	8.31(32/385)	7.61(167/2194)	13.56(96/708)	22.47(40/178)	0.606	< 0.001	< 0.001
Male factor infertility	23.64(91/385)	25.02(549/2194)	21.89(155/708)	24.71(44/178)	0.609	0.096	> 0.9
Fertilization					0.533	0.06	0.05
IVF	71.95(277/385)	73.52(1613/2194)	77.12(546/708)	80.33(143/178)			
ICSI	28.05(108/385)	26.48(581/2194)	22.88(162/708)	19.66(35/178)			

Continuous variables are expressed as mean ± SD; categorical variables are expressed as numbers (%)

a BMI < 18.5 vs. 18.5 ≤ BMI < 24. b 24 ≤ BMI < 28 vs. 18.5 ≤ BMI < 24. c BMI ≥ 28 vs.18.5 ≤ BMI < 24

**Table 2:** Embryo outcomes of each BMI groups

Parameters	BMI < 18.5	18.5 ≤ BMI < 24	24 ≤ BMI < 28	BMI ≥ 28	*P*^*a*^	*P*^*b*^	*P*^*c*^
**TOTAL: *n* = 3465**	*n* = 385	*n* = 2194	*n* = 708	*n* = 178			
Number of oocytes retrieved	9.95 ± 5.92	9.69 ± 6.53	9.94 ± 6.69	10.11 ± 6.65	0.24	0.759	0.99
Number of oocytes retrieved exclude PCOS	9.66 ± 5.87	9.46 ± 6.60	9.23 ± 6.35	8.73 ± 6.12	0.577	0.444	0.205
Mature oocytes rate (%)	83.29(3215/3860)	83.11(17922/21564)	84.06(5919/7041)	82.79(1458/1761)	0.796	0.063	0.743
IVF 2PN rate/oocytes (%)	68.51(1880/2744)	66.63(10624/15944)	67.34(3628/5387)	62.95(870/1382)	0.054	0.34	**0.006**
ICSI 2PN rate/mature oocytes (%)	72.27(628/869)	72.31(3267/4518)	70.11(936/1335)	71.12(229/322)	> 0.99	0.119	0.652
IVF (1 + ≥ 3PN)/oocytes (%)	6.41(176/2744)	7.04(1123/15944)	6.43(346/5387)	5.43(75/1382)	0.239	0.128	**0.037**
ICSI (1 + ≥ 3PN)/mature oocytes (%)	3.91(34/869)	3.65(165/4518)	3.22(43/1335)	2.48(8/322)	0.7	0.5	0.35
Total Cleavage rate (%)	98.80(2478/2508)	98.65(13703/13891)	99.01(4519/4564)	98.54(1083/1099)	0.565	0.058	0.8
IVF Cleavage rate	98.99(1861/1880)	99.08(10526/10624)	99.15(3597/3628)	98.62(858/870)	0.708	0.755	0.201
ICSI Cleavage rate	98.25(617/628)	97.25(3177/3267)	98.50(922/936)	98.25(225/229)	0.171	**0.031**	0.524
Available embryos rate (%)	58.15(1441/2478)	57.32(7854/13703)	56.61(2558/4519)	61.59(667/1083)	0.453	0.406	**0.007**
IVF Available embryos rate (%)	58.84(1095/1861)	57.62(6065/10526)	56.35(2027/3597)	62.24(534/858)	0.333	0.191	**0.009**
ICSI Available embryos rate (%)	56.08(346/617)	56.31(1789/3177)	57.59(531/922)	59.11(133/225)	0.929	0.497	0.444
Good quality embryos rate (%)	44.22(1096/2478)	43.36(5941/13703)	43.15(1950/4519)	45.89(497/1083)	0.428	0.822	0.111
IVF Good quality embryos rate (%)	45.46(846/1861)	43.59(4588/10526)	43.68(1571/3597)	46.27(397/858)	0.135	0.938	0.133
ICSI Good quality embryos rate (%)	40.52(250/617)	42.59(1353/3177)	41.11(379/922)	44.44(100/225)	0.35	0.427	0.626
Blastocyst development rate (%)	64.56(847/1312)	66.27(4602/6944)	66.14(1506/2277)	77.98(393/504)	0.24	0.919	**< 0.001**
IVF Blastocyst development rate (%)	67.66(707/1045)	67.75(3826/5647)	66.67(1248/1872)	75.43(304/403)	0.971	0.393	**0.001**
ICSI Blastocyst development rate (%)	52.43(140/267)	59.83(776/1297)	63.70(258/405)	88.12(89/101)	**0.029**	0.18	**< 0.001**
Good quality blastocyst rate (%)	21.42(281/1312)	23.62(1640/6944)	23.76(541/2277)	25.40(128/504)	0.087	0.887	0.357
IVF Good quality blastocyst rate (%)	23.35(244/1045)	24.38(1377/5647)	23.88(447/1872)	27.30(110/403)	0.504	0.686	0.188
ICSI Good quality blastocyst rate (%)	13.86(37/267)	20.28(263/1297)	23.21(94/405)	17.82(18/101)	**0.017**	0.209	0.608

Continuous variables are expressed as mean ± SD; categorical variables are expressed as numbers (%)

a BMI < 18.5 vs. 18.5 ≤ BMI < 24. b 24 ≤ BMI < 28 vs. 18.5 ≤ BMI < 24. c BMI ≥ 28 vs.18.5 ≤ BMI < 24

The normal fertilization rate of IVF cycles in the underweight group was slightly higher than in the normal group but without significant differences (all *P* > 0.05). The fertilization rate (including normal and abnormal) of IVF cycles in the obesity group was significantly decreased (normal: 62.95% vs. 66.63% *p* = 0.006; abnormal: 5.43% vs. 7.04%, *p* = 0.037). Also, compared with the normal group, the cleavage rate of ICSI cycles in the overweight group was higher (98.50% vs. 97.25%, *p* = 0.031). On D3, the available embryo rate of the obesity group was higher than that of the normal group (61.59% vs. 57.32%, *p* = 0.007), while no difference was found in the underweight and overweight groups vs. the normal group (all *p* > 0.05).

When analyzing the blastocyst stage, the blastocyst development rates and good-quality blastocyst rates of the underweight and overweight group were comparable with the normal group (all *p* > 0.05). Nevertheless, in the ICSI subgroup, the blastocyst development rate and the good quality blastocyst rate in the underweight group were lower than those in the normal group (52.43% vs. 59.83%, *p* = 0.029; 13.86% vs. 20.28%, *p* = 0.017; respectively). On the other hand, the obesity group had a higher blastocyst development rate than the normal group (77.98% vs. 66.27%, *p* < 0.001), while the good-quality blastocyst rate of this group was only slightly higher, showing no significant difference.

### Clinical and neonatal outcomes

As shown in Table 3, 1698 fresh transfer cycles were reviewed. The details of fundamental maternal characteristics of fresh transfer cycles are shown in Table S1. The number of transferred embryos, the stage of transferred embryos and at least one top-quality embryo transfer proportion were similar in each group. There were 2 and 3 cases of fetal death in the normal and overweight groups, respectively, and 1 stillbirth in each. The final lived birth number was 806. Bivariate analyses revealed no significant difference in the clinical pregnancy rate, implantation rate, abortion rate, and live birth rate. In total, 660 singletons and 146 twins were born. The gestational age of singletons born to the overweight group was lower than that of singletons born to the normal group (38.08 ± 1.95 vs. 38.95 ± 1.55, *p* = 0.011). The preterm birth rate of the obesity group was higher than the normal group (23.68% vs. 5.97%, *p* = 0.001). Fewer boys were born in overweight group than in the normal group (43.15% vs. 56.22%, *p* = 0.009). The mean birthweight of singletons in the underweight group was the lowest but did not significantly differ compared with the normal group. LBW, HBW, SGA, VSGA, and LGA of singletons were comparable among the four BMI groups. The mean birthweight of twins born in the overweight group was higher than that born in the normal group (2595.07 ± 378.53 g vs. 2417.31 ± 446.78 g, *p* = 0.007). No significant difference was observed in the remaining outcomes.

**Table 3:** Clinical and neonatal outcomes of fresh cycles in each BMI groups

Parameters	BMI < 18.5	18.5 ≤ BMI < 24	24 ≤ BMI < 28	BMI ≥ 28	*P*^*a*^	*P*^*b*^	*P*^*c*^
**TOTAL:*n* = 1698**	*n* = 188	*n* = 1066	*n* = 354	*n* = 90			
ET embryo No	1.51 ± 0.51	1.5 ± 0.51	1.48 ± 0.52	1.54 ± 0.50	0.988	0.455	0.468
Transferred embryo stage (n.)							
Cleavage	133	738	243	65	0.731	0.842	0.634
Blastocyst	55	328	111	25			
At least one top-quality embryo transferred (%)	68.09 (128/188)	66.23 (706/1066)	68.64 (243/354)	73.33 (66/90)	0.675	0.434	0.200
Endometrial thickness	11.75 ± 2.67	11.70 ± 2.53	11.82 ± 2.68	11.78 ± 2.66	0.796	0.464	0.777
Clinical pregnancy rate (%)	53.72 (101/188)	55.63 (593/1066)	59.60 (211/354)	60.0 (54/90)	0.634	0.194	0.441
Clinical pregnancy rate exclude PCOS (%)	52.57 (92/175)	55.00 (539/980)	56.27 (166/295)	59.15 (42/71)	0.565	0.739	0.538
Implantation rate (%)	42.05 (119/283)	43.73 (701/1603)	47.52 (249/524)	50.36 (70/139)	0.604	0.142	0.154
Abortion rate (%)	13.86 (14/101)	15.01 (89/593)	15.17 (32/211)	7.41 (4/54)	0.88	> 0.99	0.115
Deliveries (singleton/twins) (*n* = 806)							
singleton	74	402	146	38	0.453	0.570	0.568
twins	13	93	29	11			
Live birth rate (%)	46.28 (87/188)	46.53 (495/1066)	49.44 (175/354)	54.44 (49/90)	> 0.99	0.297	0.675
Live birth rate exclude PCOS (%)	45.14 (79/175)	45.61 (447/980)	47.46 (140/295)	54.93 (39/71)	0.934	0.594	0.140
**Singletons**							
No. of Singletons	74	402	146	38			
Gestational age (weeks)	38.75 ± 1.72	38.95 ± 1.55	38.85 ± 1.41	38.08 ± 1.95	0.307	0.508	**0.011**
Boys (%)	55.41 (41/74)	56.22 (226/402)	43.15 (63/146)	55.26 (21/38)	0.899	**0.009**	0.739
Preterm birth (< 37 weeks) rate (%)	6.76 (5/74)	5.97 (24/402)	7.53 (11/146)	23.68 (9/38)	0.792	0.554	**0.001**
Birthweight	3093.15 ± 411.64	3205 ± 478.23	3262.99 ± 490.29	3233.29 ± 529.75	0.06	0.213	0.730
Low birthweight (< 2500 g) (%)	5.41 (4/74)	5.22 (21/402)	4.79 (7/146)	7.89 (3/38)	> 0.99	> 0.99	0.451
High birthweight (> 4000 g) (%)	0 (0/74)	3.23 (13/402)	6.85 (10/146)	2.63 (1/38)	0.235	0.088	0.709
Small for gestational age (< 10th percentile)	13.51 (10/74)	8.46 (34/402)	4.79 (7/146)	0 (0/38)	0.188	0.198	0.06
Very small for gestational age (< 5th percentile)	2.70 (2/74)	4.23 (17/402)	3.42 (5/146)	0 (0/38)	0.751	0.808	0.383
Large for gestational age (> 90th percentile)	2.70 (2/74)	7.46 (30/402)	8.90 (13/146)	15.79 (6/38)	0.203	0.592	0.111
**Twins**							
No. of twins	13	93	29	11			
Gestational age (weeks)	36.08 ± 2.20	36.31 ± 1.97	36.86 ± 1.30	35.97 ± 1.40	0.694	0.16	0.585
Boys (%)	42.31 (11/26)	52.69 (98/186)	53.45 (31/58)	36.36 (8/22)	0.403	> 0.99	0.179
Preterm birth (< 37 weeks)	38.46 (5/13)	47.31 (44/93)	51.72 (15/29)	63.64 (7/11)	0.768	0.832	0.354
Birthweight (g)	2240 ± 399.40	2417.31 ± 446.78	2595.07 ± 378.53	2565.91 ± 399.22	0.056	**0.007**	0.138
Low brithwieght (< 2500 g)	57.69 (15/26)	51.61 (96/186)	37.93 (22/58)	36.36 (8/22)	0.676	0.073	0.259

Continuous variables are expressed as mean ± SD; categorical variables are expressed as numbers (%)

a BMI < 18.5 vs. 18.5 ≤ BMI < 24. b 24 ≤ BMI < 28 vs. 18.5 ≤ BMI < 24. c BMI ≥ 28 vs.18.5 ≤ BMI < 24

### Generalized linear model and binary logistic regression analyses

Considering the influence of BMI on the overweight and obesity groups was consistent, the obesity group was integrated into the overweight group to obtain more accurate statistical analysis results. We first adjusted the confounding factors when analyzing maternal BMI as a continuous variable for all cycles and three divided subgroups. The results of the generalized linear model and binary logistic regression about embryo quality, clinical and neonatal are shown in Tables 4 and 5, respectively. Among all cycles, BMI was positively correlated with the number of oocytes retrieved (Beta: 0.082, *p* = 0.029), but after excluded PCOS patients, the correlation between the two factors became insignificant (Beta: 0.032, *p* = 0.304). The beta value increased to 1.474 (*p* = 0.001) in the underweight category and was still significant after excluded PCOS patients (Beta: 0.943, 0.012) (Table 4). In addition, the blastocyst development rate was positively associated with BMI, though the beta value was low (Beta: 0.006, *p* = 0.011).

**Table 4:** Maternal BMI and associations with embryo quality

**Parameters**	**All cycles**		**BMI < 18.5**		**18.5 ≤ BMI < 24**		**24 ≤ BMI**	
	*n* = 3465		*n* = 385		*n* = 2194		*n* = 886	
	Beta(SE)	*P*	Beta(SE)	*P*	Beta(SE)	*P*	Beta(SE)	*p*
Total dose of GN	69.715 (3.761)	< 0.001	46.614 (37.028)	0.208	63.368 (9.684)	< 0.001	85.943 (11.965)	< 0.001
Number of oocytes retrieved	0.082 (0.038)	**0.029**	1.474 (0.426)	**0.001**	0.165 (0.106)	0.119	-0.098 (0.106)	0.385
Number of oocytes retrieved exclude PCOS	0.032 (0.031)	0.304	0.943 (0.374)	**0.012**	0.085 (0.083)	0.307	-0.070 (0.087)	0.421
Mature oocytes rate %	0.001 (0.001)	0.589	-0.032 (0.017)	0.064	-0.003 (0.003)	0.353	-0.01(0.004)	0.873
2PN rate	-0.001 (0.001)	0.271	-0.009 (0.016)	0.576	0.003 (0.003)	0.401	-0.001 (0.003)	0.707
1 + ≥ 3PN rate	-0.001 (0.001)	0.117	0.013 (0.009)	0.162	-0.003 (0.002)	0.112	-0.003 (0.003)	0.223
Available embryos rate (%)	-0.001(0.002)	0.706	-0.023 (0.020)	0.254	-0.011 (0.005)	**0.020**	0.003 (0.005)	0.573
Good quality embryos rate	-0.001 (0.002)	0.662	-0.017 (0.022)	0.446	-0.008 (0.005)	0.091	0.004 (0.006)	0.477
Blastocyst development rate (%)	0.006 (0.002)	**0.011**	0.028 (0.031)	0.356	-0.014 (0.006)	**0.028**	0.019 (0.006)	**0.001**
Good quality blastocyst rate (%)	0.002 (0.002)	0.232	0.005(0.023)	0.827	-0.007 (0.005)	0.179	0.005 (0.005)	0.322

Adjust for the paternal age, fertilization method (the first two results did not adjust these two confounders), maternal age, infertility type, infertility factors, infertility years, ovulating induction protocols and basal endocrine parameters (FSH, LH

**Table 5:** Maternal BMI and associations with clinical and neonatal outcomes

**Parameters**	**All cycles**		**BMI < 18.5**		**18.5 ≤ BMI < 24**		**24 ≤ BMI**	
	AOR (95% CI)	*P*	AOR (95% CI)	*P*	AOR (95% CI)	*P*	AOR (95% CI)	*P*
Clinical pregnancy rate	1.030 (0.997–1.063)	0.073	0.984 (0.632–1.534)	0.945	1.005 (0.921–1.098)	0.907	1.025(0.933–1.126)	0.602
Abortion rate	0.974 (0.918–1.033)	0.379	1.714 (0.543–5.406)	0.358	0.954 (0.811–1.121)	0.565	0.973 (0.821–1.154)	0.756
Live birth rate	1.032 (1.000–1.065)	0.053	0.944 (0.606–1.470)	0.798	0.997 (0.913–1.088)	0.938	1.048 (0.957–1.147)	0.313
**Singletons**	*N* = 660		*N* = 74		*N* = 402		*N* = 184	
Preterm birth (< 37 weeks) rate	1.134 (1.037–1.240)	**0.006**	1.634 (0.482–5.534)	0.43	1.142 (0.848–1.539)	0.382	1.43 (1.122–1.823)	**0.004**
Boys (%)	0.982 (0.947–1.102)	0.330	2.337 (0.943–5.793)	0.067	1.030 (0.976–1.088)	0.278	0.98 (0.840–1.144)	0.789
	Beta(SE)		Beta(SE)		Beta(SE)		Beta(SE)	
Gestational age (weeks)	-0.027 (0.013)	0.156	-0.092 (0.280)	0.742	0.015 (0.054)	0.778	-0.157 (0.055)	**0.004**
Birthweight	8.827 (3.398)	**0.009**	4.416 (16.629)	0.791	4.858 (5.197)	0.35	8.904 (14.152)	0.529
	AOR (95% CI)							
Low birthweight (< 2500 g) rate	0.955 (0.895–1.019)	0.163						
High birthweight (> 4000 g) rate	1.109 (0.978–1.258)	0.106						
**Twins**	*N* = 143							
Preterm birth (< 37 weeks) rate	1.074 (0.967–1.193)	0.184						
	Beta(SE)							
Gestational age (weeks)	0.028(0.044)	0.523						
Birthweight	20.549 (5.922)	**0.001**						

Adjust for: age of men and women, type of infertility, infertility factors, years of infertility, stage of transferred embryos, number of transferred embryos, whether there were top-level embryos and thickness of endometrium on the day of transfer

The effect of BMI on blastocyst development rate differed among subgroups. In the normal group, there was a negative association (Beta: -0.014, *p* = 0.028), while in the overweight group, the correlation was positive (Beta: 0.019, *p* = 0.001). Also, the BMI was negatively associated with the available embryo rate in the normal group (Beta: -0.011, *p* = 0.021). Whether in all cycles or subgroup cycles analysis, fertilization and mature oocyte rates were not associated with BMI after adjusting for confounders (all *P* > 0.05).

As shown in Table 5, when binary logistic regression was performed in clinical and neonatal analysis, the adjusted OR (AOR) of BMI for a preterm birth rate of singletons was 1.134 [(95% CI 1.037–1.240), *p* = 0.006], and the AOR value increased to 1.43 [(95% CI 1.122–1.823), *p* = 0.004] when BMI ≥ 24. BMI was not significantly associated with clinical pregnancy rate [1.030 (95% CI 0.997–1.063)], live birth rate [1.032 (95% CI 1.000–1.065)], abortion rate [0.974 (0.918–1.033)], low birthweight rate [0.955 (95% CI 0.895–1.019)], and high birthweight rate [1.109 (95% CI 0.978–1.258)] of singletons in all cycles; the results were similar in 3 BMI subgroups (Table 5). When analyzing subjects as a whole, BMI did not affect the sex ratio of neonatal [0.982 (95% CI 0.947–1.102), *p* = 0.330], but the AOR showed a tendency of a positive association between BMI and the boy ratio when BMI < 18.5 [2.337 (95% CI 0.943–5.793), *p* = 0.067]. In the generalized linear model, the relation of BMI and gestational age outcome was not significant from all cycles (Beta: -0.027, *p* = 0.156), underweight group (Beta: -0.092, *p* = 0.742) and the normal group (Beta: 0.015, *p* = 0.778), but when BMI ≥ 24, the correlation between BMI and gestational age turned significantly negative (Beta: -0.157, *p* = 0.004). Due to the limited number of twins, we only analyzed the association of preterm birth rate, gestational age, and birthweight of twins with BMI for all cycles. Maternal BMI was significantly associated with that of newborns, both in singletons (Beta: 8.827, *p* = 0.009) and twins (Beta: 20.549, *p* = 0.001). Furthermore, twin's preterm birth rate and gestational age were not associated with BMI.

Considering the interaction of PCOS and maternal age with BMI on clinical outcomes, we analyzed PCOS group (*n* = 177) and non- PCOS group (*n* = 1521), young ages (≤ 35 years) (*n* = 1346) and old ages (> 35 years) (*n* = 352) separately (Table 6). In the PCOS and > 35 years subgroups, clinical pregnancy rate, abortion rate, live birth rate, preterm birth (< 37 weeks) rate and gestational age of singletons were not associated with BMI after adjusting for confounders (all *P* > 0.05). However in non-PCOS subgroup, live birth rate and preterm birth (< 37 weeks) rate of singletons were significantly correlated with BMI, the AOR value was 1.042 [95% CI (1.007–1.078), *p* = 0.018] and 1.116 [95% CI (1.049–1.188), *p* = 0.001] respectively. In ≤ 35 years subgroup, clinical pregnancy rate, live birth rate and preterm birth (< 37 weeks) rate of singletons were positively associated with BMI, the AOR value was 1.038 [95% CI (1.001–1.076), *p* = 0.045], 1.037 [95% CI (1.002–1.074), *p* = 0.038] and 1.106 [95% CI (1.040–1.176), *p* = 0.001 respectively.

**Table 6:** BMI and associations with clinical and neonatal outcomes in different patients' subgroups

**Parameters**	PCOS		non-PCOS		≤ 35 years		> 35 years	
	AOR (95% CI)	*P*	AOR (95% CI)	*P*	AOR (95% CI)	*P*		*P*
Clinical pregnancy rate	1.046 (0.949–1.152)	0.366	1.034 (0.999–1.070)	0.054	1.038 (1.001–1.076)	**0.045**	0.992 (0.918–1.027)	0.843
Abortion rate	1.088 (0.929–1.274)	0.296	0.954 (0.894–1.018)	0.156	0.965 (0.904–1.031)	0.291	0.990 (0.863–1.135)	0.881
Live birth rate	1.013 (0.926–1.107)	0.783	1.042 (1.007–1.078)	**0.018**	1.037 (1.002–1.074)	**0.038**	1.001 (0.922–1.086)	0.986
**Singletons**								
Preterm birth (< 37 weeks) rate	0.917 (0.660–1.272)	0.602	1.116 (1.049–1.188)	**0.001**	1.106 (1.040–1.176)	**0.001**	1.137 (0.749–1.727)	0.546
	Beta(SE)		Beta(SE)		Beta(SE)		Beta(SE)	
Gestational age (weeks)	0.037 (0.062)	0.548	-0.038 (0.022)	0.083	-0.031(0.020)	0.123	0.023 (0.050)	0.643

Adjust for: age of men and women, type of infertility, infertility factors, years of infertility, stage of transferred embryos, number of transferred embryos, whether there were top-level embryos and thickness of endometrium on the day of transfer

**Fig. 1:**
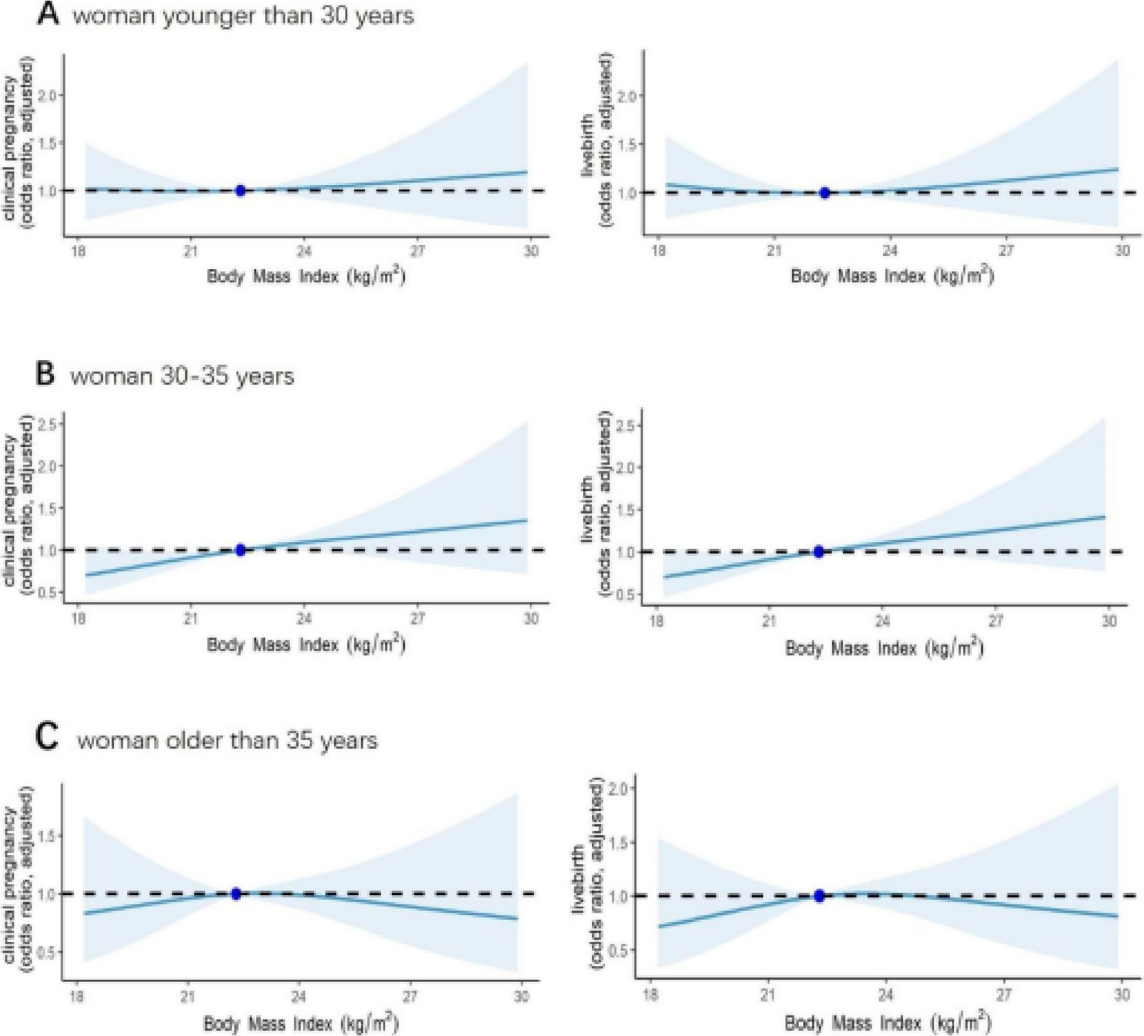
**A** non-linear logistic regression analysis of BMI and clinical pregnancy and live birth of women younger than 30. **B** non-linear logistic regression analysis of BMI and clinical pregnancy and live birth of women 30–35. **C** non-linear logistic regression analysis of BMI and clinical pregnancy and live birth of women older than 35

The analysis results of the (non-linear) logistic regression method showed that apart from the confounding factors, the effect of BMI on clinical pregnancy and live birth varied with the change in BMI (Fig. 1). In addition, this effect differed in three age groups, i.e., in women younger than 30 years, women 30–35 years old, and women older than 35 years. In those younger than 30 years, the AOR values of clinical pregnancy rate and live birth rate did not change before the BMI value reached 22.5, after which it increased. In 30–35-year-old women, the two AOR values increased with BMI, and the curve was almost linear. For women older than 35, these two AOR values increased before BMI > 22.5 and then decreased.

## Discussion

This retrospective study of southern Chinese women undergoing IVF revealed that being overweight and obese was not associated with worse embryo quality and poorer clinical outcomes in fresh transfer cycles. Many studies suggested that high BMI has a negative impact on the clinical outcome of IVF [6, 19]. In addition, for women younger than 38 years old, being overweight could reduce the cumulative live birth rate [20]. Nevertheless, the present analysis showed that with the increase in BMI, the clinical pregnancy rate, implantation rate, and live birth rate all had an upward trend. After adjusting for related confounders, including age, infertility years, infertility types, logistic regression analysis revealed similar results. For non-PCOS patients and younger women (≤ 35 years), the correlation between IVF clinical outcomes and BMI was more significant.

Many studies have shown that maternal obesity increases the risk of premature delivery [21, 22], which is consistent with our data. However, we did not find evidence for the association of miscarriage with obesity, as mentioned in a previous study [23]. Which means that the impact of BMI on pregnancy might more significant in the middle to late stages of fetal development.

Obesity is likely to cause PCOS and further impact on women's fertility. For PCOS patients, within the group, BMI did not seem to be directly related to clinical outcomes. After excluding interference from PCOS, in non-PCOS patients group, BMI positively associated with live birth rate. Similarly, the present study illustrated that BMI had more significant impact on young patients (≤ 35 years) than on old patients (> 35) which was consistent with previous reports. That might because age was not the main influencing factors for clinical outcomes for young women, the AOR of age for clinical pregnancy rate and live birth rate was 0.999 [95% CI (0.950–1.050), *p* = 0.959] and 0.981 [95% CI (0.934–1.307), *p* = 0.435] respectively. Nevertheless, the AOR value turned to 0.811 [95% CI (0.717–0.917), 0.001] and 0.775 [95% CI (0.672–0.895), *p* = 0.000] in older ages, which means age became the most significant influencing factor then. In young ages (≤ 35 years), this ananlysis found BMI was positively associated with clinical results which is at odds with the negative impact of BMI on IVF clinical outcomes reported by Sneed et al. [16]. In the previous research, sample size was smaller, and did not adjust confounders which might had impact on clinical outcomes. Further, the different patient race in the previous research might also attribute to the discrepancy.

Our results showed that the trends of the AOR curve of live birth and clinical pregnancy differed with age groups. There was a trend toward increasing the clinical pregnancy and live birth rate in an overweight group compared to the normal weight group at younger ages (≤ 35 years), but the trend was decreased in older ages (> 35). In addition, Low weight had adverse effects on patients over 30 years old, but had little impact on young ages (< 30 years). But the curve showed that older ages (> 35 years) were also susceptible to abnormal BMI. This result may be due to the interaction between age and BMI.

Overweight patients need a higher dose of Gn than normal patients to stimulate ovulation induction [24–26]. The generalized linear model showed a strong positive correlation between BMI and Gn dose, and this effect was continuous even in the normal category (18 ≤ BMI < 24). Though a relatively higher dose was used, overweight patients could still obtain a similar number of oocytes as the normal group. Considering that the retrieved oocyte number of PCOS patients would be more than normal patients, while the proportion of PCOS patients of obese patients was higher than other groups, we excluded the PCOS cycles; however, the analysis result still showed no significant difference. Some earlier studies reported that BMI would affect the quality and number of available embryos. Nevertheless, in this study, we found that the available embryo rate and blastocyst development rate in the obesity group were higher than those in the normal group. Also, another research showed that those with BMI over 24–27 had higher good embryo proportion on day 3 than those with normal weight [27].

Kim et al*.* proposed that obese patients might obtain comparable fertilization rates with those with normal BMI by ICSI [28], which we further confirmed in the present study. In the IVF group, the normal and abnormal fertilization rates in the obesity group were lower than those in the normal group; however, this phenomenon was not seen in the ICSI cycle. Nevertheless, other studies claimed that BMI has little or no effect on oocyte quality [11, 29]. Our data demonstrated that obesity does have a negative impact on oocyte fertilization, which was also the reason why the infertility years increased with BMI. Nonetheless, it seems that this impact could be avoided by changing the way of fertilization. Therefore, patients with high BMI could use ICSI to improve their fertilization rate.

None of the parameters in the underweight group embryos before day 5 differed from those in the normal group; however, the blastocyst and high-quality blastocyst development rate of ICSI cycles were significantly lower than those of the normal group. Oocytes and embryos use endogenous lipids as energy substrates for energy production, membrane components, and signaling lipids [30, 31]. The lipid content of porcine embryos changes in preimplantation development, and compared to the morula stage, it significantly decreases in blastocyst formation [32]. Endogenous lipids are mainly composed of triglycerides and cholesterol esters [31]. A previous study found that the content of triglycerides in serum and follicular fluid is higher in overweight patients than in normal-BMI patients [33]. Also, another study found that oocyte lipid droplet number is significantly correlated to fatty acid composition in follicular fluid [34]. Our follow-up analysis showed that BMI was positively correlated with the blastocyst formation rate. We speculated that BMI might affect the lipid content in oocytes and that the lipid content in women with low body weight might be relatively lower. This effect was not notable in the process of IVF fertilization. However, it was evident in ICSI cycles a certain amount of energy might be consumed for oocytes recovery after this stimulation, ultimately leading to insufficient energy support and poor blastocyst development in the later stage. Except for the obesity group, the blastocyst development rate of ICSI cycles was lower than that of IVF cycles in all other groups.

The “obesity paradox” has been reported in patients with diabetes [35], cardiovascular disease [36], osteoporosis [37], and even cancer [38]. The present study demonstrated that overweight and obese patients had comparable or even better clinical outcomes than normal patients. Similar results were obtained in the frozen-warmed embryo transfer cycles [28] but not in fresh transfer cycles before. The two studies had one thing in common: the geographical locations of reproductive centers. They had approximate latitudes and were all southeast coastal cities. In our study, patients were mainly from the southeast coastal areas of China. Environmental factors and people's living habits might be one of the reasons for this phenomenon. In China, the prevalence of obesity seems to be lower in Southeast coastal regions compared with North, Northeast, and Circum-Bohai Sea regions [39]. A large-sample, multi-center analysis demonstrated that the women with PCOS in Southern China had better clinical outcomes than Northern women following the same interventions [40]. A warmer south climate enables patients to engage in more outdoor activities and enjoy a lighter diet, all of which affect IVF outcomes. However, more studies are needed to confirm the relationship between BMI and different regions.

According to the Chinese BMI classification standard, 11.11% of patients in the present study had low body weight, 63.32% had normal body weight, 20.43% were overweight, and 5.14% were obese. Also, there were 81.46% (145/178) cases with class I obesity, 17.42% (31/178) with class II, and only 1.12% (2/178) with class III obesity. Mild obesity might also be one of the reasons for better clinical outcomes, which was also the main limitation of this study. Moreover, multi-center studies and cross-regional joint research with a larger sample size are warranted to replenish these data.

To sum up, the effect of low BMI on the development of blastocysts is worthy of attention. The present study found that pre-pregnancy overweight and obesity were not associated with poor embryonic, pregnancy, and live birth outcomes in IVF fresh transfer cycles; however, they affected blastocyst formation. ICSI could help avoid low fertilization in obese patients. Nonetheless, due to premature delivery and other hidden risks, patients’ weight should still be controlled before and during pregnancy. But the effect of age and weight loss on ART needs to be weighed.

### Supplementary Information

**Additional file 1: Supplemental Table 1.** Cycle characteristics of fresh ET cycles.

## Data Availability

The datasets generated and/or analysed during the current study are not publicly available due the datasets included the patient's medical records which were private documents, but are available from the corresponding author on reasonable request.
