# Supplementary material for: Pre-pregnancy obesity is not associated with poor outcomes in fresh transfer in vitro fertilization cycles: a retrospective study
Source: BMC Pregnancy Childbirth. 2023 Sep 2;23:633. doi: 10.1186/s12884-023-05917-7 (PMC10474631; doi:10.1186/s12884-023-05917-7)
Supplement: Supplementary file 1 — Additional file 1: Supplemental Table 1. Cycle characteristics of fresh ET cycles. [file 12884_2023_5917_MOESM1_ESM.pdf]

Supplemental Table 1. Cycle characteristics of fresh ET cycles

**Parameters**	**BMI<18.5**	**18.5≤BMI<24**	**24≤BMI<28**	**BMI≥28**	P^a	P^b	P^c
NO. of ET cycles (n.)	n=188	n=1066	n=354	n=90			
Maternal age (years)	29.98±4.22	31.64±4.81	32.01±5.10	31.94±4.28	<0.001	0.219	0.557
Infertility years	3.22±2.28	3.39±2.68	3.72±2.63	4.32±3.14	0.381	0.036	0.002
Body mass index (kg/㎡)	17.58±0.72	21.06±1.46	25.59±1.23	30.13±1.78	<0.001	<0.001	<0.001
Total dose of GN(IU)	1826.19±545.38	2074.43±674.12	2361.9±732.99	2744.36±926.73	<0.001	<0.001	<0.001
NO. of oocytes retrieved	9.66±4.81	10.29±5.23	10.81±5.72	10.58±5.77	0.535	0.112	0.604
Type of infertility					<0.001	0.057	0.585
primary infertility	70.21(132/188)	52.90(564/1066)	46.89(166/354)	48.89(44/90)			
secondary infertility	29.79(56/188)	47.09(502/1066)	53.11(188/354)	51.11(46/90)			
Main infertility factor (%)							
Tubal factor	37.23(70/188)	45.03(480/1066)	41.24(146/354)	35.55(32/90)	0.056	0.217	0.097
Endometriosis	17.55(33/188)	9.38(100/1066)	5.65(20/354)	5.56(5/90)	0.002	0.028	0.337
PCOS	6.91(13/188)	8.07(86/1066)	16.67(59/354)	21.11(19/90)	0.291	<0.001	<0.001
Male factor infertility	12.77(24/188)	13.32(142/1066)	9.89(35/354)	8.89(8/90)	0.907	0.095	0.325

Continuous variables are expressed as mean±SD; categorical variables are expressed as numbers (%).

a BMI<18.5 vs. 18.5≤BMI<24. b 24≤BMI<28 vs. 18.5≤BMI<24. c BMI≥28 vs.18.5≤BMI<24.
